# Pre-parturition staphylococcal mastitis in primiparous replacement goats: persistence over lactation and sources of infection

**DOI:** 10.1186/s13567-014-0115-6

**Published:** 2014-12-09

**Authors:** Iacome SC Jácome, Francisca GC Sousa, Candice MG De Leon, Denis A Spricigo, Mauro MS Saraiva, Patricia EN Givisiez, Wondwossen A Gebreyes, Rafael FC Vieira, Celso JB Oliveira

**Affiliations:** Department of Veterinary Sciences, Federal University of Paraiba, Areia, PB 58397-000 Brazil; Department of Animal Science, Federal University of Paraiba, Areia, PB 58397-000 Brazil; Department of Veterinary Preventive Medicine, College of Veterinary Medicine, The Ohio State University, Columbus, OH 43210 USA; Veterinary Public Health and Biotechnology Global Consortium (VPH-Biotec), The Ohio State University, Columbus, OH 43210 USA

## Abstract

This investigation reported for the first time the occurrence of intramammary infections caused by *Staphylococcus* in primiparous replacement goats before parturition and the persistence of clinical *Staphylococcus aureus* infection during the lactation period. Subclinical infections, mainly caused by coagulase negative staphylococci (CoNS), did not persist during lactation. Genotyping analysis indicated that environment seems to play a moderate role as source of intramammary infections to goats before parturition, but causative agents of mastitis in lactating animals are not genotypically related to environmental staphylococci. The occurrence and persistence of intramammary infections in replacement goats demonstrate the need to consider those animals as potential sources of infections in dairy goat herds.

## Introduction, methods, and results

In the past few years, studies demonstrating staphylococcal mastitis in replacement heifers have raised intriguing questions about the epidemiology of intramammary infection in dairy herds. This is mainly due to the fact that *Staphylococcus* has been considered a contagious agent basically transmitted among lactating animals during milking practices [1]. Recent findings suggested environment as a potential source of *Staphylococcus* causing mastitis in dairy cattle [2]. These reports clearly demonstrate that detailed knowledge on the epidemiology of intramammary infections is necessary for the establishment of control measures in order to reduce infections.

In goats, besides the fact that information about the epidemiology of mastitis is very scarce compared to dairy cattle, the occurrence of intramammary infections in replacement goats has not been reported before. Mastitis continues to be an important burden to the goat milk industry, especially in developing regions, where the goat milk production chain plays an important socioeconomic role.

Therefore, this study aimed primarily to test the hypothesis that intramammary infection does not occur in replacement goats prior to parturition. Secondly, if infection does occur, the goal was to determine the persistence of infection over lactation and to identify possible contamination sources of the pathogens on herds.

In order to test the first hypothesis, primiparous replacement goats from two farms located in the municipalities of Areia (Farm A) and Bananeiras (Farm B), Paraiba State, Brazil, were examined for clinical and subclinical mastitis. Examination for clinical mastitis in those animals included visual inspection of the udder and colostrum in order to detect definitive abnormalities, such as blood clots. For subclinical mastitis investigation, bacteriological culture was performed in 26 colostrum samples from half (one teat) of the udders of seven animals in Farm A (AP 1–7) and six in Farm B (BP 1–6) within 15 days before parturition. Samples were aseptically collected and processed according to a reference protocol [3]. Before sampling procedures, teats were disinfected with iodine pre-milking solution, dried with disposable paper towels and disinfected using cotton balls moist with 70% ethyl alcohol. Samples were kept refrigerated and processed within 6 h after samplings. The identification of any mastitis agent by isolation approach in any of the animals was considered enough to accept or refute the initial hypothesis.

Persistence of pre-parturition infections over lactation was investigated using a follow-up approach by means of clinical examination and milk samplings performed in all primiparous goats two times after parturition (at 30 and 60 days after first sampling). As performed for colostrum samplings, pre-dipping using iodine solution, drying with disposable paper towel and disinfection with 70% ethyl alcohol were performed before milk samples were collected for microbiological analysis. Fore-milk was discarded and a strip-cup test was used to detect signs of clinical mastitis.

In order to detect potential sources of infection to primiparous replacement goats, three multiparous goats from Farm A (AM 1–3) and five from Farm B (BM 1–5) were included in the follow-up trial. Prepartum colostrum samples (*n* = 16) were collected from multiparous goats in the first sampling.

Swabs from teats (*n* = 63), and nostrils (*n* = 63) were collected from the primiparous and multiparous goats, as well as environmental samples, including milking restraint devices (*n* = 6), stalls (*n* = 6) and milking room wall (*n* = 6), and swabs from the hand of milkers (*n* = 6) from both farms in the three samplings. Samples were collected using Stuart agar gel medium transport swabs (product # 141C, Copan, USA), which were kept under refrigeration until microbiological analyses. Finally, milk samples (*n* = 84) were collected from the lactating primiparous and multiparous animals in the second and third samplings.

Bacteriological isolation was performed by streaking a loopful of milk samples onto blood agar (product # 7166A, Blood agar base no.2, Acumedia, USA, enriched with 5% sheep blood) and MacConkey agar (product # 212123, BD Difco, USA) plates. Positive cultures were identified by means of Gram staining, morphological characteristics and biochemical tests. Gram positive cocci were tested for catalase and oxidase production. Confirmed staphylococci were tested for production of coagulase in tubes. In the present study, we considered intramammary infections when milk or colostrum samples from half udders cultured positive for 4 or more non-hemolytic or one or more hemolytic colony forming units.

*Staphylococcus* species from milk and colostrum from primiparous and multiparous goats were identified by a microplate biochemical panel (Combo PC33, Siemens Healthcare, USA) using a semi-automated system (Autoscan 4, Siemens Healthcare).

Genotyping by Rep-PCR was performed to identify possible transmission routes of mastitis-causing agents in both farms. Genomic DNA was extracted [4] and 25-μL reactions contained Taq DNA polymerase buffer (1X), MgCl_2_ (3 mM), Taq DNA polymerase (1 U; Invitrogen, USA), dNTPs (200 μM each; Ludwing Biotec, Brazil), primer RW3A [5] (1 pMol; Invitrogen, USA), and DNA template (100 ng). Amplification cycles included 1 min at 94 °C, 1 min at 50 °C, and 2 min at 72 °C. Dendograms were built by an unweighted pair group method with arithmetic mean clustering algorithm (UPGMA) and the genetic similarity between isolates was calculated using the Dice coefficient (SD), using Bionumerics v. 7.1 (Applied Maths, Belgium). Clusters of isolates were assigned using 80% genetic similarity as a cutoff. The discriminatory index (DI value) of Rep-PCR was calculated as reported [6].

The frequency of staphylococci positive samples in the different sample sources collected from Farms A and B is presented in Table 1. The microbiological culture results of prepartum colostrum and milk samples collected in the three samplings performed in Farms A and B are shown in Table 2. Bacteria were recovered from 15 of the 126 (11.9%) milk and colostrum samples, from which five originated from Farm A and ten from Farm B. All isolates were staphylococci and the majority were CoNS. Amongst the five positive samples from Farm A, *S. haemolyticus* (*n* = 5) and *S. epidermidis* (*n* = 1) were identified. Positive samples for staphylococci included swabs of teats (*n* = 25), nostrils (*n* = 26), hands (*n* = 2), device (*n* = 3), stall (*n* = 2) and wall (*n* = 3). In Farm B, staphylococci were isolated from four primiparous goats and one multiparous goat, including *S. aureus* (*n* = 5), *S. hyicus* (*n* = 2), *S. epidermidis* (*n* = 1), *S. auricularis* (*n* = 1), *S. cohnii* (*n* = 1)*, S. hominis* (*n* = 1), *S. xylosus* (*n* = 1). Positive samples for staphyloccoci were also identified in swabs of teats (*n* = 27), nostrils (*n* = 26), hands (*n* = 3), device (*n* = 3), stall (*n* = 1) and wall (*n* = 3).

**Table 1:** **Frequency of** ***Staphylococcus*** **sp. in different sample sources taken from two small-scale goat milk production systems in Paraiba, Northeastern Brazil**

**Sample source**	**Farm A**				**Farm B**			
	**1** ^**st**^ **(Prepartum)**	**2** ^**nd**^	**3** ^**rd**^	**Total**	**1** ^**st**^ **(Prepartum)**	**2** ^**nd**^	**3** ^**rd**^	**Total**
Prepartum colostrum*	1/10	-	-	1/10	4/11	-	-	4/11
Milk	-	1/10	1/10	2/20	-	2/11	4/11	6/22
Teat swabs	9/10	7/10	9/10	25/30	9/11	8/11	10/11	27/33
Nostril swabs	9/10	8/10	9/10	26/30	8/11	8/11	10/11	26/33
Milkers’ hand swabs	0/1	1/1	1/1	2/3	1/1	1/1	1/1	3/3
Milking restraint device	1/1	1/1	1/1	3/3	1/1	1/1	1/1	3/3
Stall	1/1	0/1	1/1	2/3	1/1	0/1	0/1	1/3
Wall of milking room	1/1	1/1	1/1	3/3	1/1	1/1	1/1	3/3

*Only prepartum animals were sampled.

**Table 2:** ***Staphylococcus*** **spp. isolated from prepartum colostrum (1** ^**st**^ **sampling) and milk samples (2** ^**nd**^ **and 3** ^**rd**^ **samplings) collected from primiparous replacing goats (AP and BP) and multiparous goats (AM and BM) in Paraiba, Northeastern Brazil**

**Sampling source**	**Sampling order**		
	**1** ^**st**^ **(Prepartum)**	**2** ^**nd**^	**3** ^**rd**^
**Farm A**			
AP1	-	-	-
AP2	-	-	-
AP3	-	-	-
AP4	*S. haemolyticus* ^1^	*S. epidermidis*	*-*
AP5	-	-	-
AP6	-	-	*-*
AP7	*-*	-	-
AM1	-	-	*S. haemolyticus* ^2^
AM2	-	-	-
AM3	-	-	-
**Farm B**			
BP1	-	-	-
BP2	*S. aureus; S. hyicus* ^3^	*S. aureus*	*S. aureus*
BP3	*S. aureus* ^2^	*S. hyicus*	*S. epidermidis*
BP4	-	-	-
BP5	-	-	*S. xylosus*
BP6	*S. cohnii*	-	-
BM1	-	-	-
BM2	-	-	-
BM3	-	-	-
BM4	*S. auricularis*	-	*S. hominis*
BM5	-	-	-

^1^ *S. haemolyticus* isolated from right and left half udders; ^2^One isolate from each half udder; ^3^ *S. aureus* from left half udder and *S. hyicus* from right half udder.

Clinical mastitis was detected in one half udder of a primiparous goat (BP2) from Farm B in all three samplings. *S. aureus* was isolated from the prepartum colostrum and post-partum milk samplings. None of the other animals (12 primiparous and 8 multiparous goats) showed clinical mastitis. Subclinical intramammary infection (*S. haemolyticus*) was detected in the prepartum colostrum of both half udders of a replacement goat from Farm A (AP4). Interestingly, *S. epidermidis* was isolated in the second sampling from a half udder previously infected by *S. haemolyticus*. In Farm B, two prepartum colostrum samples from primiparous goats were positive for *S. hyicus* (BP3) and *S. cohnii* (BP6). Besides, *S. auricularis* was isolated from prepartum colostrum from a multiparous goat (BM4) in this herd. In this farm, different CoNS species were also cultured from the same half udder in consecutive samplings, such as *S. hyicus* and *S. epidermidis* isolated from the right half udder of a primiparous goat (BP3) in the second and third samplings, respectively. *S. auricularis* and *S. hominis* were isolated from the same half udder in the first and third samplings of a multiparous goat (BM4).

**Figure 1:**
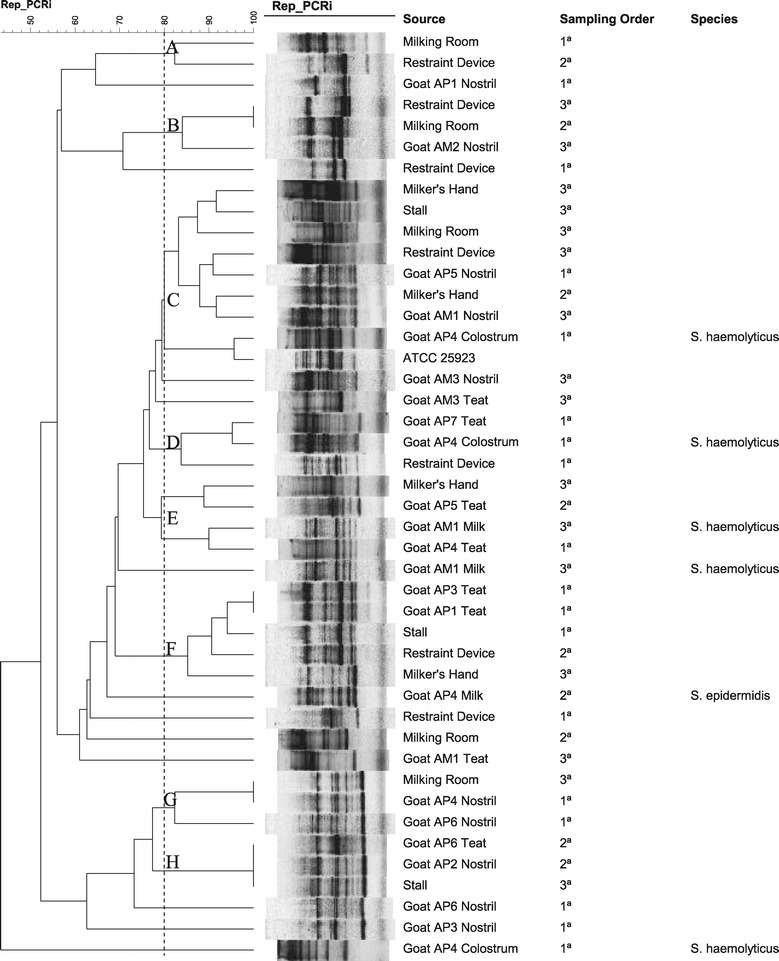
**Genotypic relatedness of staphylococci from a dairy goat herd.** Rep-PCR dendrogram illustrating the genotypic relatedness of staphylococci isolated from different sample sources in a small-scale goat milk production system (Farm A) in Paraiba State, Northeastern Brazil, which shows eight clusters **(A-H)** at 80% similarity among the band profiles.

Rep-PCR showed DI values of 0.99 (Farm A) and 0.98 (Farm B). Staphylococci isolates from Farm A were assigned to eight distinct clusters (A to H) and 12 non-clustered isolates (Figure 1). Interestingly, no clonal relatedness amongst *S. haemolyticus* from colostrum (AP4) and milk (AM1) was detected. Two *S. haemolyticus* isolated from prepartum colostrum (AP4) showed fingerprints similar to those staphylococci cultured from environment, teat surfaces and nostrils of other goats and hands (clusters C and D). In the majority of the clusters (B, C, D, F, G, and H), isolates from teat surface and nostrils were highly related to those from environment and in some instances showed indistinguishable patterns (clusters G and H).

**Figure 2:**
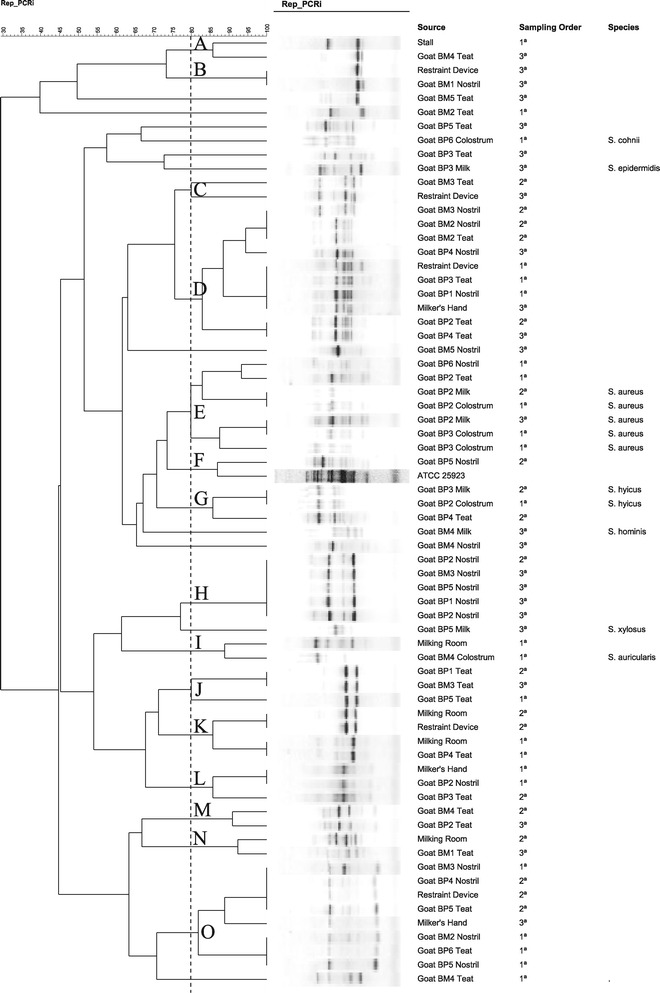
**Genotypic relatedness of staphylococci from a dairy goat herd.** Rep-PCR dendrogram illustrating the genotypic relatedness of staphylococci isolated from different sample sources in a small-scale goat milk production system (Farm B) in Paraiba State, Northeastern Brazil, which shows fifteen clusters **(A-O)** at 80% similarity among the band profiles.

In farm B, fifteen clusters (A to O) of staphylococci were identified (Figure 2). One half udder of BP2 was infected in the first and second samplings by *S. aureus* sharing indistinguishable genotypic patterns (Figure 2). Some staphylococci from environment sources and hands showed undistinguished fingerprints compared with isolates from nostrils and teat surfaces from primiparous and multiparous goats (clusters B, D, K, L and O). *S. auricularis* cultured from colostrum of a multiparous goat (BM4) was related to an isolate from the wall (cluster I). Interestingly, CoNS isolated from mastitic milk, such as *S. epidermidis* (BP3), *S. hominis* (BM4), and *S. xylosus* (BP5) were not clustered with isolates from environment sources. Cluster E was comprised exclusively by *S. aureus* with clonally related isolates infecting one half udder (BP2) in different samplings. This cluster included no isolates from environment.

## Discussion

The recovery of *S. aureus* from the same clinically infected udder in primiparous replacement goats before and after parturition suggest that this agent can persist over the lactation period. This assumption is reinforced since the isolates showed indistinguishable fingerprinting patterns. On the other hand, it seems not to be the case for subclinical intrammamary infections caused by the *S. aureus* (BP3) and CoNS species. This is because no species causing subclinical infections were cultured from a given infected udder in consecutive samplings. Moreover, the shift in the etiology of CoNS infections, as observed in AP4, suggests that subclinical infection caused by those organisms in primiparous replacement goats did not persist over the lactation period. Our results in goats corroborate previous findings observed in dairy cattle, suggesting that subclinical mastitis by CoNS is milder, has a shorter-term persistence and is associated to a higher spontaneous cure rate compared to *S. aureus* [7].

It is important to recognize the possible limitations of the semi-automated system used in the present study in identifying CoNS accurately, especially if we consider rare or infrequent species from animal origin. Therefore, assumptions on the putative transmission pathways of CoNS in the investigated farms were taken based on the findings of the Rep-PCR fingerprinting patterns. Rep-PCR was able to differentiate epidemiologically related isolates of the same *Staphylococcus* species. Besides, the high DI values indicate that Rep-PCR is a useful tool in typing staphylococci from goat farms. Rep-PCR using RW3A has been successfully used to discriminate epidemiologically related *S. aureus* of animal [5,8] and human origins [9].

The similar genotypic patterns of *S. haemolyticus* from prepartum colostrum (AP4) and environment in Farm A suggest that udder infections in the prepartum period might possibly be associated to environmental sources, even because no isolate from mastitis during lactation was related to environmental staphylococci. This assumption is reinforced by the fact that *S. haemolyticus* from prepartum colostrum of the same animal (AP4) showed a different DNA fingerprint pattern compared to *S. haemolyticus* cultured from milk from a multiparous goat in the same farm (AM1).

The fact that *S. auricularis* cultured from colostrum of a multiparous goat (BM4) was genotypically related to an isolate from the milking room (cluster I, Figure 2) suggest environment as the potential source of prepartum intramammary infection*.* Considering the similar results obtained for *S. haemolyticus* in Farm A, we believe that environment plays an important role as a source of intramammary infection for CoNS species in the prepartum period.

It is noteworthy that the genotypic patterns of some *Staphylococcus* isolated from nostrils and teat surface of primiparous goats in the prepartum period had indistinguishable DNA fingerprint patterns to isolates that originated from the wall of the milking room (cluster K). However, primiparous goats had no access to the milking room before lactation implying an independent source of contamination with the same strain. This finding corroborates with the earlier statement on *S. auricularis* isolated from a prepartum colostrum showing identical genotypic pattern with one isolated from the wall of the milking room (cluster I). The similar genotypic patterns of isolates from milkers’ hand swabs, environment, and animals (nostrils and teat surface), as shown in clusters D, L, and O, might indicate the widespread dissemination of clonally related staphylococci in dairy goat milk production systems. Milkers could play a role on the dissemination of staphylococci, since isolates from the surface of teats of primiparous goats (non-lactating animals) were clonally related to isolates that originated from milkers’ hand swabs (Figure 2, cluster D). Since primiparous and multiparous goats share the same environment in the prepartum period in dairy goat production systems in Northeastern Brazil, this continuous flow farming system facilitates the spread of microorganisms across the different groups. Recent reports in cows demonstrated a similar finding indicating higher risk of intramammary infections in heifers raised in contact to multiparous cows with the same mastitis causing agents involved [1,10].

Despite the high similarity amongst staphylococci from environmental sources and body surfaces of goats, our results indicated that those organisms were not the causal agents of intramammary infections in the primiparous lactating goats. The absence of environmental isolates clustered with *S. aureus* (Cluster E, Figure 2) strongly suggests *S. aureus-*associated intramammary infections to be contagious but environmental dissemination occurs rarely. Our study indicates that the epidemiology of mastitis caused by CoNS is complex in goats, which is expected, since this group is represented by a large range of species with different pathogenic traits. Indeed, CoNS are the most frequent mastitis-causing agents in lactating goats [8,11-13].

In conclusion, clinical and subclinical intramammary infections in primiparous replacement goats can occur in the prepartum period and can persist over the lactation if caused by *S. aureus* in the clinical form. While environment seems to play a role as source of intramammary infections to goats before parturition, causative agents of mastitis in lactating animals are not genotypically related to staphylococci of environmental origin. The occurrence and persistence of intramammary infections in replacement goats demonstrated herein indicate the need to consider testing the replacement animals and take necessary precaution and preventive measures to avoid spreading of mastitis-causing staphylococci as they remain potential sources of infections in goat dairy herds. Longitudinal studies investigating the extent of CoNS species as causative agents of intramammary infections in the goat species are recommended.
